# Amyloid and tau PET-positive cognitively unimpaired individuals are at high risk for future cognitive decline

**DOI:** 10.1038/s41591-022-02049-x

**Published:** 2022-11-10

**Authors:** Rik Ossenkoppele, Alexa Pichet Binette, Colin Groot, Ruben Smith, Olof Strandberg, Sebastian Palmqvist, Erik Stomrud, Pontus Tideman, Tomas Ohlsson, Jonas Jögi, Keith Johnson, Reisa Sperling, Vincent Dore, Colin L. Masters, Christopher Rowe, Denise Visser, Bart N. M. van Berckel, Wiesje M. van der Flier, Suzanne Baker, William J. Jagust, Heather J. Wiste, Ronald C. Petersen, Clifford R. Jack, Oskar Hansson

**Affiliations:** 1 Lund University, Clinical Memory Research Unit, Lund, Sweden; 2 Alzheimer Center Amsterdam, Neurology, Vrije Universiteit Amsterdam, Amsterdam UMC location VUmc, Amsterdam, the Netherlands; 3 Amsterdam Neuroscience, Neurodegeneration, Amsterdam, the Netherlands; 4 Memory Clinic, Skåne University Hospital, Malmö, Sweden; 5 Department of Radiation Physics, Skåne University Hospital, Lund, Sweden; 6 Skåne University Hospital, Department of Clinical Physiology and Nuclear Medicine, Lund, Sweden; 7 Department of Neurology, Massachusetts General Hospital, Harvard Medical School, Boston, MA USA; 8 Center for Alzheimer Research and Treatment, Department of Neurology, Brigham and Women’s Hospital, Harvard Medical School, Boston, MA USA; 9 Athinoula A. Martinos Center for Biomedical Imaging, Department of Radiology, Massachusetts General Hospital, Harvard Medical School, Boston, MA USA; 10 Department of Radiology, Massachusetts General Hospital, Harvard Medical School, Boston, MA USA; 11 The Australian e-Health Research Centre CSIRO Melbourne Victoria Australia, Melbourne, Victoria Australia; 12 Department of Molecular Imaging & Therapy Austin Health Melbourne Victoria Australia, Melbourne, Victoria Australia; 13 The Florey Institute of Neuroscience and Mental Health Melbourne Victoria Australia, Parkville, Victoria Australia; 14 Department of Radiology and Nuclear Medicine, Vrije Universiteit Amsterdam, Amsterdam UMC location VUmc, Amsterdam, the Netherlands; 15 Department of Epidemiology and Biostatistics, Vrije Universiteit Amsterdam, Amsterdam UMC location VUmc, Amsterdam, the Netherlands; 16 Lawrence Berkeley National Laboratory, Berkeley, CA USA; 17 Helen Wills Neuroscience Institute, UC Berkeley, Berkeley, CA USA; 18 Department of Quantitative Health Sciences, Mayo Clinic, Rochester, MN USA; 19 Department of Neurology, Mayo Clinic, Rochester, MN USA; 20 Department of Radiology, Mayo Clinic, Rochester, MN USA

**Keywords:** Prognostic markers, Alzheimer's disease

## Abstract

A major unanswered question in the dementia field is whether cognitively unimpaired individuals who harbor both Alzheimer’s disease neuropathological hallmarks (that is, amyloid-β plaques and tau neurofibrillary tangles) can preserve their cognition over time or are destined to decline. In this large multicenter amyloid and tau positron emission tomography (PET) study (*n* = 1,325), we examined the risk for future progression to mild cognitive impairment and the rate of cognitive decline over time among cognitively unimpaired individuals who were amyloid PET-positive (A^+^) and tau PET-positive (T^+^) in the medial temporal lobe (A^+^T_MTL_^+^) and/or in the temporal neocortex (A^+^T_NEO-T_^+^) and compared them with A^+^T^−^ and A^−^T^−^ groups. Cox proportional-hazards models showed a substantially increased risk for progression to mild cognitive impairment in the A^+^T_NEO-T_^+^ (hazard ratio (HR) = 19.2, 95% confidence interval (CI) = 10.9–33.7), A^+^T_MTL_^+^ (HR = 14.6, 95% CI = 8.1–26.4) and A^+^T^−^ (HR = 2.4, 95% CI = 1.4–4.3) groups versus the A^−^T^−^ (reference) group. Both A^+^T_MTL_^+^ (HR = 6.0, 95% CI = 3.4–10.6) and A^+^T_NEO-T_^+^ (HR = 7.9, 95% CI = 4.7–13.5) groups also showed faster clinical progression to mild cognitive impairment than the A^+^T^−^ group. Linear mixed-effect models indicated that the A^+^T_NEO-T_^+^ (*β* = −0.056 ± 0.005, *T* = −11.55, *P* < 0.001), A^+^T_MTL_^+^ (*β* = −0.024 ± 0.005, *T* = −4.72, *P* < 0.001) and A^+^T^−^ (*β* = −0.008 ± 0.002, *T* = −3.46, *P* < 0.001) groups showed significantly faster longitudinal global cognitive decline compared to the A^−^T^−^ (reference) group (all *P* < 0.001). Both A^+^T_NEO-T_^+^ (*P* < 0.001) and A^+^T_MTL_^+^ (*P* = 0.002) groups also progressed faster than the A^+^T^−^ group. In summary, evidence of advanced Alzheimer’s disease pathological changes provided by a combination of abnormal amyloid and tau PET examinations is strongly associated with short-term (that is, 3–5 years) cognitive decline in cognitively unimpaired individuals and is therefore of high clinical relevance.

## Abstract (web summary)

Abnormal amyloid and tau PET in cognitively unimpaired individuals is strongly associated with short-term cognitive decline and subsequent development of dementia.

## Main

**Fig. 1:**
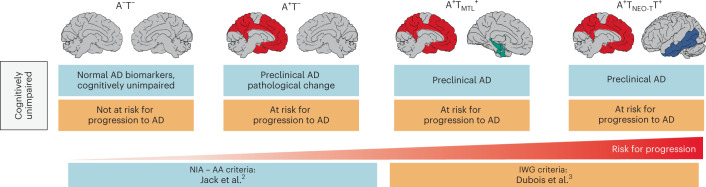
NIA-AA versus IWG criteria. Differences in the nomenclature of cognitively unimpaired individuals with (^+^) or without (−) in vivo biomarker evidence of Aβ (A) and tau (T) pathology in the NIA-AA versus IWG criteria for AD. Note that for the IWG criteria, the presumed ‘risk for progression’ level rises when both A and T biomarkers are positive.

Alzheimer’s disease (AD) is neuropathologically characterized by the presence of amyloid-β (Αβ) plaques and tau neurofibrillary tangles. Although the two most well-established diagnostic criteria for AD both acknowledge the importance of Αβ and tau pathology in AD pathogenesis^1^, an important distinction is that the National Institute on Aging and Alzheimer’s Association (NIA-AA) criteria^2^ define AD by its biological features (that is, the presence of Αβ and tau pathology) irrespective of the clinical syndrome, whereas the International Working Group (IWG) criteria^3^ require the presence of objective cognitive impairment (mild cognitive impairment (MCI) or dementia) in conjunction with positive AD biomarkers. Consequently, there is fundamental disagreement between the criteria about the nomenclature for cognitively unimpaired individuals who harbor one or both AD hallmark neuropathological features. For example, a cognitively unimpaired individual with positive Αβ (Α^+^) and tau (T^+^) biomarkers is classified as ‘preclinical AD’ by the NIA-AA criteria^2^, while the IWG criteria^3^ would label such an individual ‘at risk for progression to AD’ (Fig. 1).

Aside from philosophical differences (for example, according to the IWG criteria the term AD should be restricted to symptomatic individuals), the discrepancy between the NIA-AA and IWG can be explained by at least two factors. First, according to the IWG criteria, currently available Αβ and tau biomarkers show ‘low predictive accuracy’ for development of cognitive symptoms. For Αβ biomarkers alone this may indeed be the case^4,5^, although PET studies with long follow-ups (approximately 10 years) indicate substantial Αβ-associated risk for cognitive decline^6^, dementia^7^ and death^7^. For tau biomarkers, however, the clinicopathological correlates are much stronger than for Αβ biomarkers^8,9^, even in asymptomatic individuals^10,11^. Second, most prognostic studies to date are based on cerebrospinal fluid (CSF) biomarkers of soluble phospho-tau levels, which is an early marker of AD pathology^12,13^. In contrast, the more recently introduced tau PET technique measures more advanced pathological changes since it captures insoluble tau aggregates^14,15^. Although tau PET positivity in the neocortex is relatively rare among cognitively unimpaired individuals (approximately 5–10%^16–18^), its presence is strongly associated with worse cross-sectional^10,19–22^ and longitudinal^11,23–32^ cognitive outcomes. However, large-scale longitudinal studies with combinations of amyloid PET and tau PET biomarker profiles as predictors of clinical progression among cognitively unimpaired individuals are lacking^33^.

In addition to addressing the differences between the NIA-AA and IWG criteria, early detection of AD pathological changes may be key for future interventions with disease-modifying treatments since these will most likely be cost-effective and show the most favorable benefit versus risk ratio when specifically targeting preclinical populations with AD that are most likely to experience substantial cognitive deterioration in the short term (that is, 3–5 years). Therefore, the aim of the current multicenter study was to examine clinical progression to MCI or dementia and assess cognitive decline in cognitively unimpaired individuals with different Αβ (A) and tau (T) biomarker profiles as defined by PET at baseline. We divided A^+^T^+^ individuals into medial temporal lobe (MTL) only (A^+^T_MTL_^+^) and temporal neocortical (A^+^T_NEO-T_^+^) T^+^ groups to additionally investigate the impact of more advanced tau pathological changes on clinical progression.

## Results

### Participants

**Extended Data Fig. 1:**
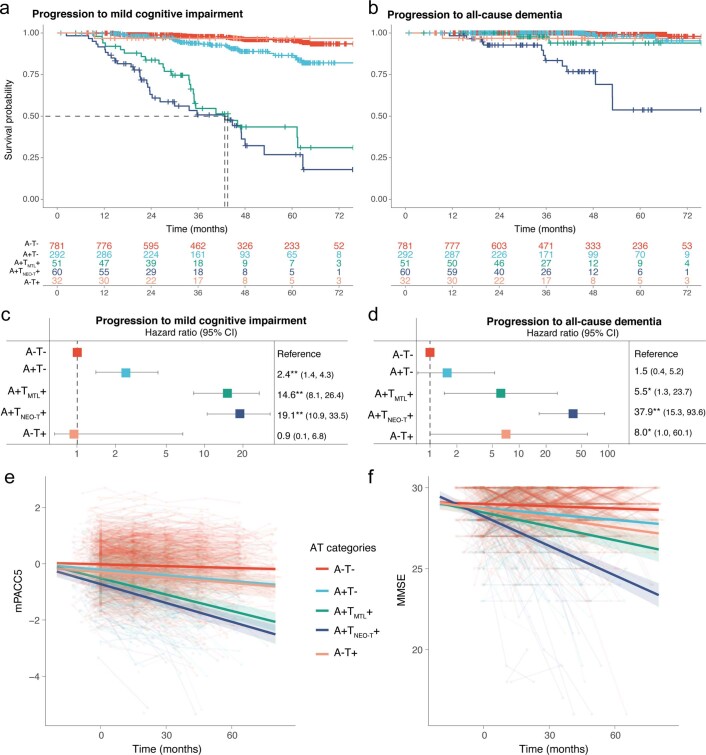
Main analyses from Figs. 2 and 3, but now also including the A-T + group. This figure resembles parts of Figs. 2 and 3 of the main manuscript but now also includes the A-T + group. **a**, Survival curves in relation to progression to MCI in the different AT categories, with the table of total number of participants available at each time point. **b**, Survival curves in relation to progression to all-cause dementia in the different AT categories, with the table of total number of participants available at each time point. **c, d** Forest plots showing the hazard ratios and 95% confidence intervals from the survival analyses shown in a and b, from Cox regression models including age, sex, education, and cohort as covariates. Statistical tests were two-sided without adjustment for multiple comparisons. **e**, Cognitive trajectories of mPACC5 scores over time in the different AT categories **f**, Cognitive trajectories of MMSE scores over time in the different AT categories. The average regression line for each group was plotted from linear mixed effect models including age, sex, education, and cohort as covariates. The error bands correspond to the 95% confidence interval. Data are anchored to the tau-PET visit (Time 0), and cognitive data up to 1 year prior to PET was included. *p = 0.01, **p < 0.001.

We included 1,325 cognitively unimpaired participants from 7 cohorts, of whom 843 (63.6%) were A^−^T^−^, 328 (24.8%) A^+^T^−^, 55 (4.2%) A^+^T_MTL_^+^ and 65 (4.9%) A^+^T_NEO-T_^+^ (see Table 1 and Supplementary Table 1 for a breakdown by cohort). All biomarker-positive groups were older and had lower baseline Mini-Mental State Examination (MMSE) scores compared to the A^−^T^−^ group (all *P* < 0.001). There were no sex differences between groups. The average follow-up duration was 41.8 ± 18.9 months. The A^−^T^+^ group was considerably smaller than the other groups (*n* = 34; Extended Data Table 1), hence their results are only reported in Extended Data Fig. 1.

**Table 1:** Participant characteristics

	A^−^T^−^	A^+^T^−^	A^+^T_MTL_^+^	A^+^T_NEO-T_^+^	*P*
*n*	843	328	55	65	
Age, years	68.6 ± 9.6	75.5 ± 8.2	75.6 ± 6.6	76.4 ± 6.8	<0.001^a^
Sex, *n* (%) male	424 (50.3)	164 (50.0)	24 (43.6)	31 (47.7)	0.822
Education, years	14.7 ± 3.1	14.6 ± 3.1	13.7 ± 3.8	13.9 ± 3.5	0.02^b^
Follow-up duration, months	43.0 ± 18.9	40.1 ± 17.6	39.0 ± 16.5	36.4 ± 14.9	0.004^c^
Follow-up visits, number	4.1 ± 1.5	4.1 ± 1.4	4.0 ± 1.3	3.6 ± 1.2	0.07
MMSE, baseline score	29.0 ± 1.0	28.7 ± 1.3	28.3 ± 1.5	28.2 ± 1.3	<0.001^d^
Progression to MCI, *n* (%)	26 (8.9%)	26 (3.3%)	25 (49.0%)	32 (53.3%)	<0.001
Progression to all-cause dementia, *n* (%)	4 (0.5%)	3 (1.0%)	2 (3.9%)	12 (20.0%)	<0.001

*P* values from two-sided statistical tests were reported. ANOVAs were used for continuous variables and chi-squared tests were used for categorical variables. Post-hoc tests were adjusted using Bonferroni correction.

^a^A^+^T_NEO-T_^+^ and A^+^T_MTL_^+^ and A^+^T^−^ > A^−^T^−^^b^ post-hoc tests revealed no significant group differences, ^c^A^−^T^−^ > A^+^T_NEO-T_^+^, ^d^A^+^T_NEO-T_^+^, A^+^T_MTL_^+^ and A^+^T^−^ < A^−^T^−^ and A^+^T_NEO-T_^+^ < A^+^ T^-^.

### Clinical progression to MCI

**Fig. 2:**
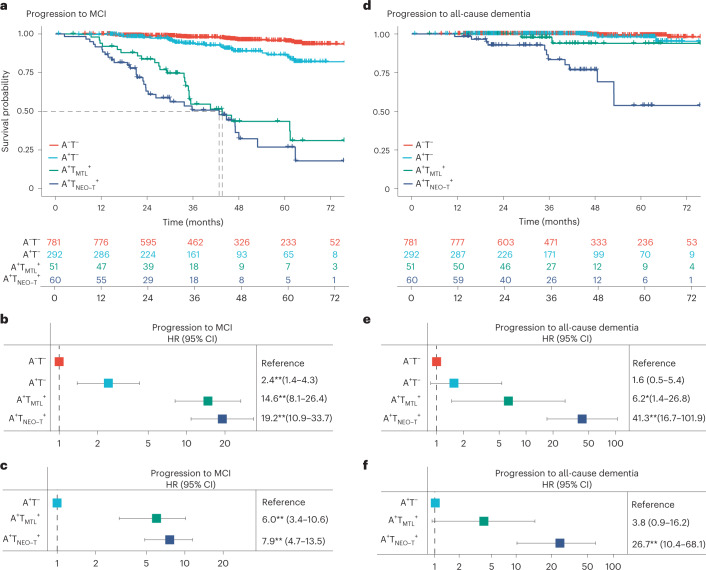
Progression to MCI or all-cause dementia in the different AT biomarker profiles. **a**,**d**, Survival curves for progression to MCI (**a**) or all-cause dementia (**d**) in the different AT biomarker profiles (A^−^T^+^: *n* = 292; A^+^T_MTL_^+^: *n* = 51; A^+^T_NEO-T_^+^: *n* = 60) with the A^−^T^−^ group (*n* = 781) as the reference, including a table of total number of participants available at each time point. The dashed line in **a** indicates the time point at which 50% of a group had progressed to MCI. **b**,**e**, Forest plots showing the HRs and 95% CIs derived from the survival analyses shown in **a** (b) and **d**(e), from Cox regression models including age, sex, education and cohort as covariates. **c**,**f**, Forest plots showing the HRs and 95% CIs derived from Cox regression models including age, sex, education and cohort as covariates but now using the A^+^T^−^ group as the reference with the outcome being progression to MCI (**c**) and progression to all-cause dementia (**f**). Statistics from two-sided tests without adjustment for multiple comparisons are reported. *P < 0.01, **P < 0.001.

During the clinical follow-up, 26 out of 781 (3.3%) of A^−^T^−^, 26 out of 292 (8.9%) of A^+^T^−^, 25 out of 51 (49.0%) of A^+^T_MTL_^+^ and 32 out of 60 (53.3%) of A^+^T_NEO-T_^+^ participants progressed to MCI or dementia. Among A^+^T_NEO-T_^+^ individuals, the progressors (27.8 ± 1.5) had worse baseline MMSE scores compared to stable individuals (28.6 ± 1.0, *P* = 0.02; Extended Data Table 2) and tended to have higher tau PET retention at baseline (Supplementary Fig. 1), suggesting that progressors were already in a slightly more advanced disease stage at the start of this study. Cox proportional-hazards models, adjusted for age, sex, education and cohort, showed an increased risk for future progression to MCI in the A^+^T_NEO-T_^+^ (HR = 19.2, 95% CI = 10.9–33.7, *P* < 0.001), A^+^T_MTL_^+^ (HR = 14.6, 95% CI = 8.1–26.4, *P* < 0.001) and A^+^T^−^ (HR = 2.4, 95% CI = 1.4–4.3, *P* = 0.002) groups compared to the A^−^T^−^ (reference) group (Fig. 2a,b). Both A^+^T_MTL_^+^ (HR = 6.0, 95% CI = 3.4–10.6, *P* < 0.001) and A^+^T_NEO-T_^+^ (HR = 7.9, 95% CI = 4.7–13.5, *P* < 0.001) groups also showed faster clinical progression to MCI than the A^+^T^−^ group (Fig. 2c). Pairwise log-rank tests showed that the A^+^T_MTL_^+^ and A^+^T_NEO-T_^+^ groups did not differ from each other (*P* = 0.19). Fifty percent of the A^+^T_NEO-T_^+^ and A^+^T_MTL_^+^ groups had progressed to MCI after 42.8 and 43.6 months, respectively.

### Clinical progression to all-cause dementia

**Extended Data Fig. 2:**
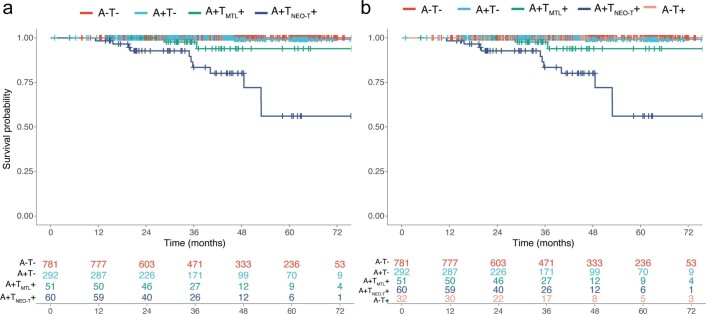
Progression to AD dementia in the different AT categories. **a**, Survival curves in relation to progression to AD dementia in the main AT categories, with the table of total number of participants available at each time point. **b**, Survival curves in relation to progression to AD dementia when including the A^-^T ^+^ group, with the table of total number of participants available at each time point. Given the small number of events, the hazard ratios are difficult to interpret, but the A + T-, A + T_MTL_ + and A + T_NEO-T_ + groups had higher HR’s compared to A-T- reference group (all p < 0.001). Pairwise comparisons indicated that both the A + T_NEO-T_ + (p < 0.001) and A + T_MTL_ + (p = 0.008) groups differed from the A + T- group, and the A + T_NEO-T_ + group differed from the A + T_MTL_ + group (p = 0.01).

During clinical follow-up, 21 participants progressed to all-cause dementia: 4 out of 781 (0.5%) in A^−^T^−^, 3 out of 292 (1.0%) in A^+^T^−^, 2 out of 51 (3.9%) in A^+^T_MTL_^+^ and 12 out of 60 (20%) in A^+^T_NEO-T_^+^. Of those, 14 progressed to clinically defined AD-type dementia and 7 to non-AD dementias (see Extended Data Table 3 for dementia type). Cox proportional-hazards models, adjusted for age, sex, education and cohort, demonstrated an increased risk for future progression to all-cause dementia in the A^+^T_NEO-T_^+^ (HR = 41.3, 95% CI = 16.7–101.9, *P* < 0.001) and A^+^T_MTL_^+^ (HR = 6.2, 95% CI = 1.4–26.8, *P* = 0.01) groups compared to the A^−^T^−^ (reference) group (Fig. 2d,e). There was no difference between the A^+^T^−^ and the A^−^T^−^ group (HR = 1.6, 95% CI = 0.5–5.4, *P* = 0.53). The A^+^T_NEO-T_^+^ (HR = 26.7, 95% CI = 10.4–68.1, *P* < 0.001) group showed faster clinical progression to all-cause dementia than the A^+^T^−^ group, while there was no significant difference between the A^+^T_MTL_^+^ (HR = 3.8, 95% CI = 0.9–16.2, *P* = 0.08) and the A^+^T^−^ group (Fig. 2f). Pairwise log-rank tests showed that the A^+^T_NEO-T_^+^ group progressed significantly faster to all-cause dementia than the A^+^T_MTL_^+^ group (*P* = 0.01). Similar results were found when using progression to AD-type dementia (11 A^+^T_NEO-T_^+^, 2 A^+^T_MTL_^+^ and 1 A^+^T^−^) as outcome instead of all-cause dementia (Extended Data Fig. 2).

### Cognitive trajectories

**Fig. 3:**
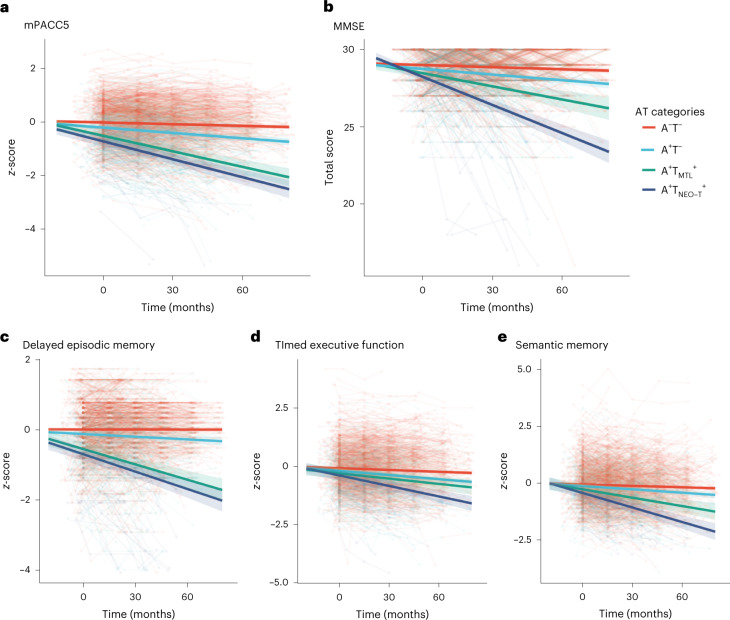
Longitudinal cognitive decline in the different AT biomarker profiles. **a**–**e**, Cognitive trajectories of mPACC5 (**a**), MMSE (**b**) and subcomponents of the mPACC5 including delayed episodic memory (**c**), timed executive function (**d**) and semantic memory (**e**) over time in the different AT biomarker profiles. The average regression line for each group was plotted from linear mixed-effect models including age, sex, education and cohort as covariates. Data are anchored to the tau PET visit (time 0); cognitive data up to 1 year before PET was included. The error bands correspond to the 95% CI.

Linear mixed-effect models adjusting for age, sex, education and cohort indicated that the A^+^T_NEO-T_^+^ (standardized *β* (std*β*) of interaction with time in months ± s.e. = −0.020 ± 0.002, *T* = −10.14, *P* < 0.001), A^+^T_MTL_^+^ (std*β* = −0.017 ± 0.002, *T* = −8.84, *P* < 0.001) and A^+^T^−^ (std*β* = −0.005 ± 0.001, *T* = −5.26, *P* < 0.001) groups showed faster decline over time on the modified preclinical Alzheimer cognitive composite 5 (mPACC5 (ref.^34^)) compared to the A^−^T^−^ (reference) group (Fig. 3a). Additionally, the A^+^T_NEO-T_^+^ (std*β* = −0.16 ± 0.002, *T* = −7.53, *P* < 0.001) and A^+^T_MTL_^+^ (std*β* = −0.13 ± 0.002, *T* = −6.21, *P* < 0.001) groups progressed faster than the A^+^T^−^ group but there was no difference between the A^+^T_NEO-T_^+^ and A^+^T_MTL_^+^ groups (std*β* = −0.003 ± 0.002, *T* = −1.13, *P* = 0.26). Exploratory study of the mPACC5 subcomponents showed that A^+^T_NEO-T_^+^ and A^+^T_MTL_^+^ groups did not differ on delayed episodic memory (std*β* = −0.002 ± 0.003, *P* = 0.47; Fig. 3c) but the A^+^T_NEO-T_^+^ group showed faster decline on timed executive function (std*β* = −0.007 ± 0.002, *P* = 0.003; Fig. 3d) and semantic memory (std*β* = −0.009 ± 0.003, *P* = 0.007; Fig. 3e).

**Extended Data Fig. 3:**
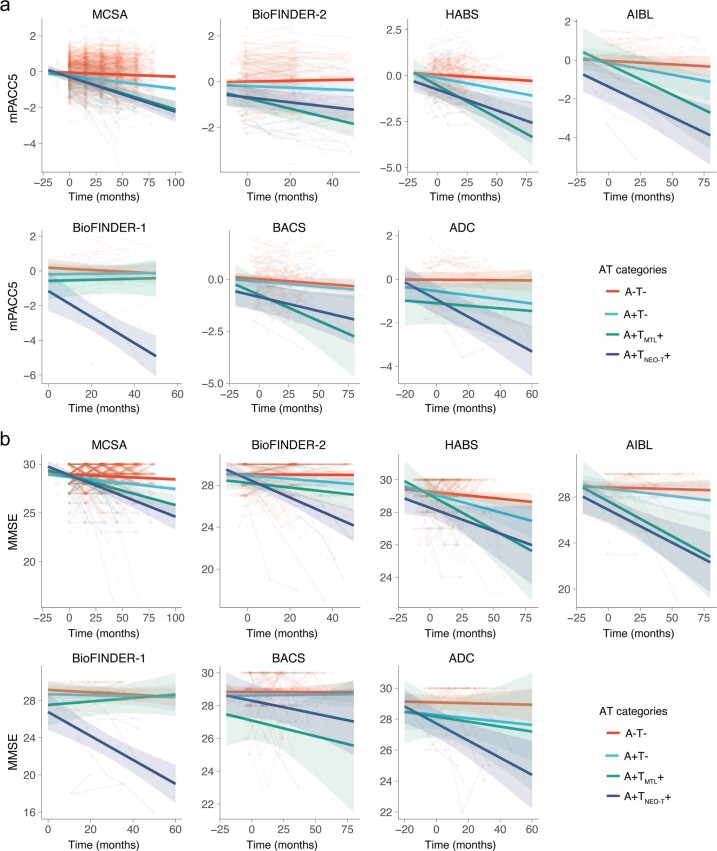
Cognitive decline on mPACC5 and MMSE in the individual cohorts. **a**, Cognitive trajectories of mPACC5 scores over time in the different AT categories in each individual cohort. **b**, Cognitive trajectories of MMSE scores over time in the different AT categories in each individual cohort. The average regression line for each group was plotted from linear mixed effect models including age, sex, and education as covariates. The error bands correspond to the 95% confidence interval. Data are anchored to the tau-PET visit (Time 0), and cognitive data up to 1 year prior to PET was included.

On the MMSE, the A^+^T_NEO-T_^+^ (*β* = −0.056 ± 0.005, *T* = −11.55, *P* < 0.001), A^+^T_MTL_^+^ (β = −0.024 ± 0.005, *T* = −4.72, *P* < 0.001) and A^+^T^−^ (*β* = −0.008 ± 0.002, *T* = −3.46, *P* < 0.001) groups showed faster decline over time compared to the A^−^T^−^ (reference) group (Fig. 3b). The A^+^T_NEO-T_^+^ (std*β* = −0.49 ± 0.005, *T* = −9.51, *P* < 0.001) and A^+^T_MTL_^+^ (std*β* = −0.16 ± 0.005, *T* = −3.04, *P* = 0.002) groups progressed faster than the A^+^T^−^ group and the A^+^T_NEO-T_^+^ group declined faster than the A^+^T_MTL_^+^ group (std*β* = −0.033 ± 0.007, *T* = −4.82, *P* < 0.001). Cognitive trajectories on the MMSE and mPACC5 for each cohort are displayed in Extended Data Fig. 3.

### Replication across different age groups

Based on the lower age of the A^−^T^−^ group compared to the biomarker-positive groups (Table 1), we performed the Cox proportional-hazards models and linear mixed models (1) stratified by different age groups (that is, 50–69, 70–79 and 80+ years; Tables 2 and 3) and (2) restricting the A^−^T^−^ group to individuals >65 years so that groups were age-matched (Extended Data Tables 4 and 5). All analyses yielded highly similar results as the primary analyses, suggesting that age did not explain the observed differences in clinical progression rates between AT groups and that the findings can be generalized across different age groups.

**Table 2:** Survival analyses across different age groups

	All ages A^−^T^−^: *n* = 781 A^+^T^−^: *n* = 292 A^+^T_MTL_^+^: *n* = 51 A^+^T_NEO-T_^+^: *n* = 60	50–69 years A^−^T^−^: *n* = 433 A^+^T^−^: *n* = 75 A^+^T_MTL_^+^: *n* = 12 A^+^T_NEO-T_^+^: *n* = 10	70–79 years A^−^T^−^: *n* = 256 A^+^T^−^: *n* = 123 A^+^T_MTL_^+^: *n* = 25 A^+^T_NEO-T_^+^: *n* = 29	80+ years A^−^T^−^: *n* = 92 A^+^T^−^: *n* = 94 A^+^T_MTL_^+^: *n* = 14 A^+^T_NEO-T_^+^: *n* = 21
	HR (95% CI), *P*	HR (95% CI), *P*	HR (95% CI), *P*	HR (95% CI), *P*
**Progression to MCI**				
A^+^T^−^	2.43 (1.38–4.26), *P* = 0.002	0.94 (0.25–3.59), *P* = 0.93	2.93 (1.16–7.39), *P* = 0.02	2.41 (0.90–6.42), *P* = 0.08
A^+^T_MTL_^+^	14.60 (8.06–26.41), *P* < 0.001	17.19 (5.98–49.42), *P* < 0.001	21.94 (8.51–56.56), *P* < 0.001	5.14 (1.39–18.94), *P* = 0.01
A^+^T_NEO-T_^+^	19.19 (10.93–33.71), *P* < 0.001	17.21 (4.54–65.19), *P* < 0.001	26.07 (10.79–62.99), *P* < 0.001	11.28 (3.91–32.57), *P* < 0.001
**Progression to all-cause dementia**				
A^+^T^−^	1.58 (0.46–5.41), *P* = 0.47	2.50 (0.27–23.00), *P* = 0.42	1.33 (0.16–10.84), *P* = 0.78	0.90 (0.50–15.98), *P* = 0.94
A^+^T_MTL_^+^	6.22 (1.44–26.79), *P* = 0.01	18.09 (1.99–165), *P* = 0.01	NA	6.26 (0.34–116), *P* = 0.22
A^+^T_NEO-T_^+^	41.26 (16.70–101.93), *P* < 0.001	28.17 (4.47–177), *P* < 0.001	47.62 (9.55–237), *P* < 0.001	27.69 (2.49–307.56), *P* = 0.007

The HR are derived from Cox proportional-hazards models with clinical progression (progression to MCI or all-cause dementia) as outcome, age, sex, education and cohort as covariates and A^−^T^−^ serving as the reference group.

NA, there were no progressors to dementia in the A^+^T_MTL_^+^ group for this particular age bin.

**Table 3:** Linear mixed models across different age groups

	All ages A^−^T^−^: *n* = 843 A^+^T^−^: *n* = 328 A^+^T_MTL_^+^: *n* = 55 A^+^T_NEO-T_^+^: *n* = 65	50–69 years A^−^T^−^: *n* = 442 A^+^T^−^: *n* = 77 A^+^T_MTL_^+^: *n* = 12 A^+^T_NEO-T_^+^: *n* = 10	70–79 years A^−^T^−^: *n* = 284 A^+^T^−^: *n* = 148 A^+^T_MTL_^+^: *n* = 27 A^+^T_NEO-T_^+^: *n* = 33	80+ years A^−^T^−^: *n* = 117 A^+^T^−^: *n* = 103 A^+^T_MTL_^+^: *n* = 16 A^+^T_NEO-T_^+^: *n* = 22
	Std*β* (s.e.), *P*	Std*β* (s.e.) *P*	Std*β* (s.e.) *P*	Std*β* (s.e.) *P*
**mPACC5**				
A^+^T^−^	−0.005 (0.001), *P* < 0.001	−0.002 (0.001), *P* = 0.11	−0.004 (0.001), *P* = 0.005	−0.003 (0.003), *P* = 0.33
A^+^T_MTL_^+^	−0.017 (0.002), *P* < 0.001	−0.015 (0.003), *P* < 0.001	−0.016 (0.003), *P* < 0.001	−0.015 (0.005), *P* = 0.004
A^+^T_NEO-T_^+^	−0.020 (0.002), *P* < 0.001	−0.025 (0.004), *P* < 0.001	−0.019 (0.003), *P* < 0.001	−0.012 (0.005), *P* = 0.02
**MMSE**				
A^+^T^−^	−0.008 (0.002), *P* < 0.001	−0.001 (0.003), *P* = 0.73)	−0.007 (0.004), *P* = 0.06	−0.013 (0.007), *P* = 0.06
A^+^T_MTL_^+^	−0.024 (0.005), *P* < 0.001	−0.039 (0.008), *P* < 0.001)	−0.006 (0.008), *P* = 0.43	−0.032 (0.014), *P* = 0.02
A^+^T_NEO-T_^+^	−0.056 (0.005), *P* < 0.001	−0.050 (0.011), *P* < 0.001	−0.049 (0.007), *P* < 0.001	−0.063 (0.013), *P* < 0.001

The std*β* values are derived from the linear mixed models adjusted for age, sex, education and cohort and represent the group × time interaction with the A^−^T^−^ serving as the reference group. *P* values from two-sided statistical tests without adjustment for multiple comparisons are reported.

### Findings are independent of white matter lesions

To account for the potential effect of white matter lesions on clinical progression, we additionally adjusted the Cox proportional-hazards models and linear mixed models for white matter hypointensity volumes (Extended Data Tables 6 and 7). These analyses yielded highly similar results as the primary analyses, suggesting that the observed differences in clinical progression rates between AT groups were independent of white matter pathology.

## Discussion

To examine whether amyloid and tau PET-positive cognitively unimpaired individuals are destined to decline, we performed a multicenter study in 1,325 participants with, on average, approximately 3.5 years of clinical follow-up data available. We found that A^+^T_NEO-T_^+^ and A^+^T_MTL_^+^ cognitively unimpaired individuals had clearly increased risk for future development of MCI and all-cause dementia and showed steep trajectories of cognitive decline. Hence, evidence of advanced AD pathology provided by amyloid and tau PET is strongly associated with short-term clinical progression in initially cognitively unimpaired individuals. This supports the NIA-AA criteria-based classification of A^+^T^+^ cognitively unimpaired individuals as ‘preclinical AD’, especially when ‘T’ is defined by PET. To consider A^+^T^+^ merely as a risk factor, and not manifest disease, may be an underestimation of its malignancy.

Although the A^+^T_NEO-T_^+^ group was at increased risk for progression to all-cause dementia compared to the A^+^T_MTL_^+^ group, there were no differences in risk for progression to MCI. A potential explanation is that tau pathological changes in the MTL can cause severe-enough memory loss leading to an individual being classified as MCI (but not dementia), while widespread tau pathology into the neocortex might be needed to produce a dementia syndrome^16,17,35^. Supporting this hypothesis, we found that the A^+^T_NEO-T_^+^ group exhibited faster decline in global cognition, semantic memory and timed executive function but not in delayed episodic memory function compared to the A^+^T_MTL_^+^ group (Fig. 3c–e).

Another important finding was the higher clinical progression rate for both A^+^T^+^ groups compared to the A^+^T^−^ group, with HRs up to 7.9 and 26.7 for progression to MCI and all-cause dementia, respectively. This indicates that among A^+^ cognitively unimpaired individuals, who are presumably already on the AD pathological continuum, the coexistence of tau pathological changes in the MTL and/or the neocortex represents a ssubstantial additional relative risk for short-term cognitive decline. However, a proportion of the A^+^T^+^ group remained cognitively intact after approximately 3.5 years of follow-up, highlighting the variable rates of cognitive decline even among individuals who are at the highest risk of deterioration based on their AD biomarker profile^5–7,23,24,36^. This possibly represents highly resilient individuals due to a favorable genetic makeup and/or a healthy lifestyle^37–39^. Alternatively, these nonprogressors may harbor fewer additional factors on top of Aβ and tau pathology, for example, synaptic loss, copathologies or neuroinflammation, resulting in an attenuation of their cognitive decline^40–42^. Hence, research into both resilience and risk factors is necessary to optimize future prediction models.

The main strengths of this study include the large-scale multicenter dataset with available amyloid PET, tau PET and longitudinal clinical and cognitive data, which allowed us to accurately estimate the relative risk of the different AT groups in terms of clinical progression and cognitive decline. This study also has several limitations. First, there are inherent challenges due to the multicenter study design, such as data pooling and harmonization, different amyloid and tau PET tracers and dissimilarities in PET acquisition protocols. Also, different neuropsychological tests were used as mPACC5 subcomponents across cohorts and these differences could impact the overall mPACC5 score. Relatedly, the varying rates of clinical progression in the A^+^T_NEO-T_^+^ and A^+^T_MTL_^+^ groups across cohorts (Supplementary Table 1) could be due to chance given the relatively small number of T^+^ participants but might also be a result of different ascertainment and recruitment methods. Second, the number of events (that is, progression to MCI and especially dementia) was relatively low. Third, we may have underestimated the actual risk of A^+^T_NEO-T_^+^ and A^+^T_MTL_^+^ cognitively unimpaired individuals due to consent or volunteer bias (lower study participation among individuals at risk) and informative censoring (the tendency of people to drop out when experiencing onset or worsening of symptoms)^43^. Fourth, we acknowledge that our design only allowed establishing relative risk and not lifetime risk and we did not control for the competing risk of death in our survival analyses.

Future studies should test whether our findings are generalizable to more diverse populations in terms of ethnicity, socioeconomic status and medical comorbidities. Furthermore, studies with longer follow-ups and larger samples of A^+^T_NEO-T_^+^ and A^+^T_MTL_^+^ cognitively unimpaired individuals will help refine the current findings. This may, in turn, aid in enriching clinical trials for fast progressors and developing algorithms for a personalized prognosis that reliably estimates the risk for future cognitive decline at an individual level. Of particular interest will be the performance of head-to-head studies between tau PET and high performing plasma p-tau assays^44–47^ to investigate the potential added value and cost-effectiveness of tau PET as a prognostic tool.

## Methods

### Participants

We included 1,325 participants from the Mayo Clinic Olmsted Study of Aging^48^ (MCSA, *n* = 680), the Swedish BioFINDER-1 (*n* = 56) and BioFINDER-2 (*n* = 228) studies at Lund University^10,16^, the Berkeley Aging Cohort Study^49^ (BACS, *n* = 109), the Harvard Aging Brain Study^50^ (HABS, *n* = 162, data obtained in March 2022 from data release 2.0 via https://habs.mgh.harvard.edu), the Australian Imaging, Biomarker & Lifestyle Flagship Study of Ageing^51^ (AIBL, *n* = 48) and the SCIENCe project^52^, which is part of the Amsterdam Dementia Cohort (ADC, *n* = 42). A brief description of each cohort is provided in Supplementary Table 2. All participants were (1) cognitively unimpaired at baseline defined by neuropsychological test scores within the normative range given an individual’s age, sex and educational background, (2) had amyloid PET available to determine Αβ status, (3) underwent a tau PET scan before 1 January 2019, to allow for sufficiently long follow-up and (4) had at least one clinical follow-up visit available. Follow-up data were collected until 1 April 2022. Amyloid and tau PET scans included in the study were acquired at the same time point in most cases and always within a maximum of 1 year of each other. Written informed consent was obtained from all participants and local institutional review boards for human research approved the study. This includes the Mayo Clinic and Olmsted Medical Center institutional review boards for MSCA, regional ethics committee at Lund University for BioFINDER-1 and BioFINDER-2, institutional review boards at Lawrence Berkeley National Laboratory and the University of California, Berkeley, for BACS, institutional human research ethics committees of Austin Health, St. Vincent’s Health, Hollywood Private Hospital and Edith Cowan University for AIBL, partners human research committee for HABS and the medical ethics review committee of the Amsterdam University Medical Center for ADC.

### Amyloid PET status

Amyloid PET scans were performed and analyzed at each respective cohort site. Αβ status was determined using center-specific cutoffs or visually read metrics using [^18^F]flutemetamol PET for BioFINDER-1 and BioFINDER-2, [^11^C]Pittsburgh compound-B PET for MCSA, BACS and HABS, [^18^F]florbetapir PET for ADC and AIBL (*n* = 47 out of 48) and [^18^F]NAV4694 for AIBL (*n* = 1 out of 48). Each cohort provided the Αβ status for their participants (see Supplementary Table 3 for details).

### Tau PET status

Tau PET was performed using [^18^F]flortaucipir across all cohorts, except BioFINDER-2 where [^18^F]RO948 was used, and data were processed according to previously described procedures (Supplementary Table 4). BioFINDER-1, BioFINDER-2 and BACS (part of a previous multicenter study^23^) tau PET scans were analyzed at Lund University. For the other cohorts, tau PET scans were processed at the respective sites; standardized uptake value ratios and region-of-interest (ROI) volumes were sent to the statistical analysis team (R.O. and A.P.B.) at Lund University. Based on these data, we computed the tau PET status for an MTL (an unweighted average of bilateral entorhinal cortex and amygdala) and an NEO-T (a weighted average of bilateral middle temporal and inferior temporal gyri) ROI. We used an unweighted MTL ROI because we intended the entorhinal cortex to have a relatively higher contribution since this region is involved in the earliest stages of tau accumulation yet it is somewhat smaller compared to the amygdala. The MTL and NEO-T ROIs were modified from a previously described temporal meta-ROI^17,53^ based on a well-established stereotypical progression of tau pathology from the MTL into the lateral temporal cortex^19,54,55^. The threshold was determined for each cohort separately, based on the mean + 2 × s.d. across all Αβ-negative participants in each cohort (see cohort-specific cutoffs in Supplementary Table 4). Based on amyloid and tau PET status, we generated four different biomarker groups: A^−^T^−^; A^+^T^−^; A^+^T_MTL_^+^ (defined as tau PET-positive in the MTL but not in the neocortex); and A^+^T_NEO-T_^+^ (defined as tau PET-positive in the NEO-T and/or MTL; 49 out of 65 were also T_MTL_^+^). The A^−^T^+^ group was considerably smaller than the other groups (*n* = 34; Extended Data Table 1), hence their results are only reported in Extended Data Fig. 1.

### Clinical outcome measures

We used both binary and continuous measures of clinical progression. First, we examined progression from cognitively unimpaired to MCI (Fig. 2a), all-cause dementia (Fig. 2d) or AD-type dementia (Extended Data Fig. 2). MCI was established using the Petersen criteria^56^ and is defined as significant cognitive symptoms as assessed by a physician, in combination with cognitive impairment on one or multiple domains (for example, memory, executive functioning, attention, language) that is below the normative range given an individual’s age, sex and educational background but not sufficiently severe to meet the diagnostic criteria for dementia. A large systematic review assessing 11,000 studies showed convergence across practices when validated diagnostic tools were used, such as in the current study^57^. AD-type dementia was diagnosed using established criteria^58^. Both MCI and dementia diagnoses were made by clinicians who were blinded for any PET or CSF outcome. For BACS, no formal diagnosis of MCI or dementia was made during the study; hence, the cohort was excluded from this analysis. Second, we examined cognitive trajectories using a sensitive composite measure specifically developed to detect cognitive changes in preclinical stages of AD (that is, the mPACC5 (refs. ^34,59^); Fig. 3a) and a screening tests of global cognition (that is, the MMSE, which is frequently used in clinical practice and in trials; Fig. 3b). The mPACC5 consists of tests capturing episodic memory, executive function, semantic memory and global cognition^34^. Individual tests were *z*-transformed using the baseline test scores of Αβ-negative participants in each cohort as the reference group and then averaged to obtain a composite *z*-score. The composition of mPACC5 is described for each cohort in Supplementary Table 5. Note that we used a modified version of the mPACC5 because, although we measured the same cognitive domains, the specific tests used in this study are not consistent with the original mPACC5 and they differ by cohort. However, a direct comparison between the original PACC5 and the mPACC5 in HABS (where the original PACC5 was developed) showed a strong correspondence between the 2 versions (*r* = 0.89, *P* < 0.001; Supplementary Fig. 2).

### Statistical analyses

All statistical analyses were performed in R v.4.0.5. Differences in baseline characteristics between groups were assessed using analysis of variance (ANOVA) with post-hoc *t*-tests with Bonferroni correction for continuous variables and chi-squared and Kruskal–Wallis with post-hoc Mann–Whitney *U*-tests for categorical or ordinal variables. First, we examined progression from cognitively unimpaired to MCI (Fig. 2a), all-cause dementia (Fig. 2d) or AD-type dementia (Extended Data Fig. 2) using Cox proportional-hazards models, adjusting for age, sex, education and cohort using A^−^T^−^ as the reference group. We additionally repeated the Cox proportional-hazards model analysis while using the A^+^T^−^ group as the reference group. Furthermore, we compared the A^+^T_MTL_^+^ and A^+^T_NEO-T_^+^ groups using pairwise log-rank tests with false discovery rate correction. For individuals who progressed to MCI and subsequent dementia, we used the respective times at conversion to MCI and dementia for the analyses presented in Fig. 2a,d. Second, we examined differences in cognitive trajectories between groups on the mPACC5 (Fig. 3a) and on global cognition (that is, the MMSE; Fig. 3b) using linear mixed-effect models with random intercepts and slopes, adjusting for age, sex, education and cohort. Finally, we examined whether our findings would generalize across different age groups and whether our results would be consistent when accounting for white matter pathology. Therefore, we performed two sets of analyses. First, we performed the main analyses when (1) stratifying the participants into different age groups (that is, 50–69, 70–79 and 80+ years old) and (2) restricting the A^−^T^−^ group to individuals older than 65 years, so that all groups were age-matched. Second, we additionally adjusted the statistical models for a measure of white matter pathology, that is, white matter hypointensity volumes derived from T1-weighted magnetic resonance imaging scans using the standard FreeSurfer pipeline^60^. Statistical significance for all models was set at two-sided *P* < 0.05.

### Reporting summary

Further information on research design is available in the Nature Research Reporting Summary linked to this article.

## Online content

Any methods, additional references, Nature Research reporting summaries, source data, extended data, supplementary information, acknowledgements, peer review information; details of author contributions and competing interests; and statements of data and code availability are available at 10.1038/s41591-022-02049-x.

### Supplementary information

Supplementary Information Supplementary Tables 1–5 and Figs. 1 and 2.

Reporting Summary

## Figures and Tables

**Extended Data Table 1:**
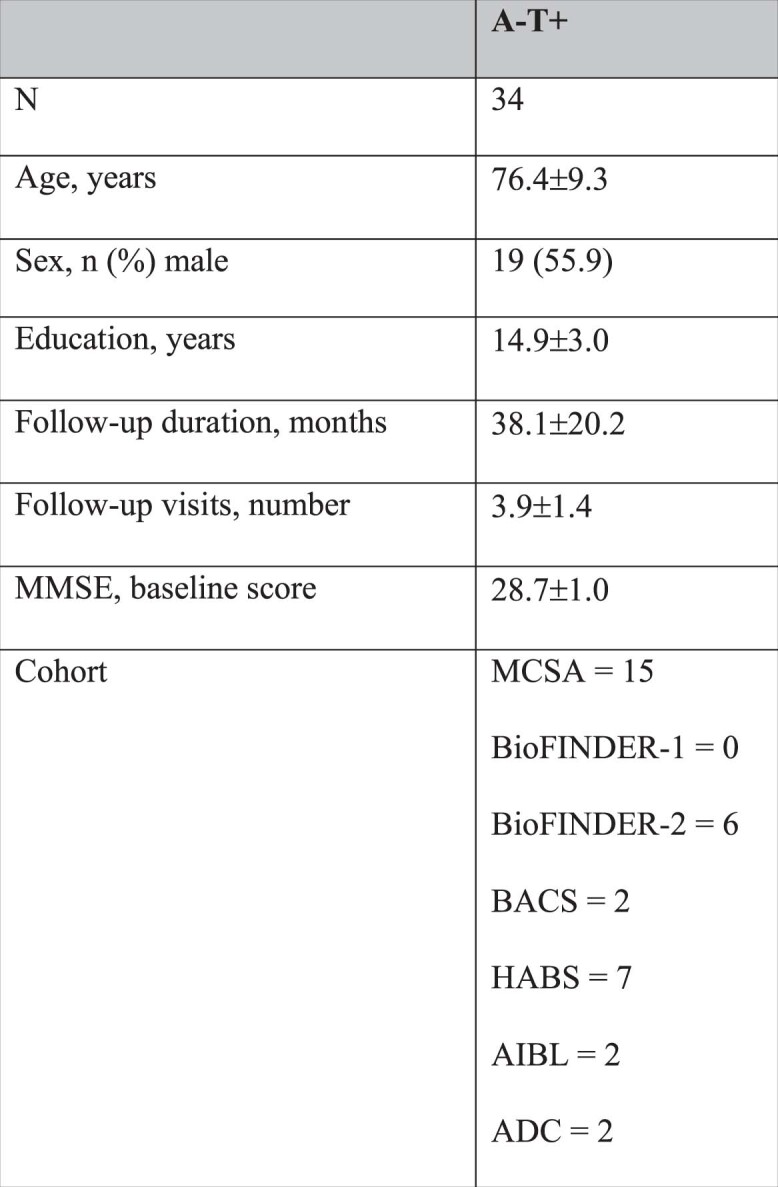
Participant characteristics of the A^−^T^+^ group

**Extended Data Table 2:**
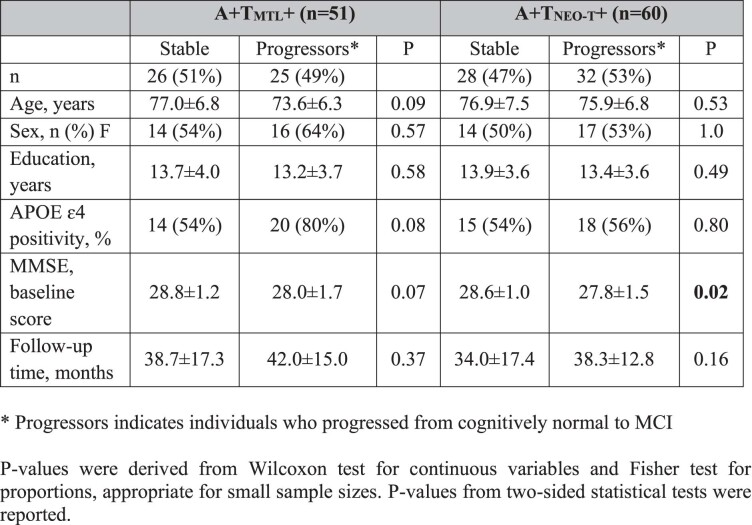
Characterization of stable individuals versus progressors in A−TMTL+ and A−TNEO-T+ groups. Progressors indicates individuals who progressed from cognitively normal to MCI. P values were derived from a Wilcoxon test for continuous variables and Fisher test for proportions, appropriate for small sample sizes. P values from two-sided statistical tests were reported

**Extended Data Table 3:**
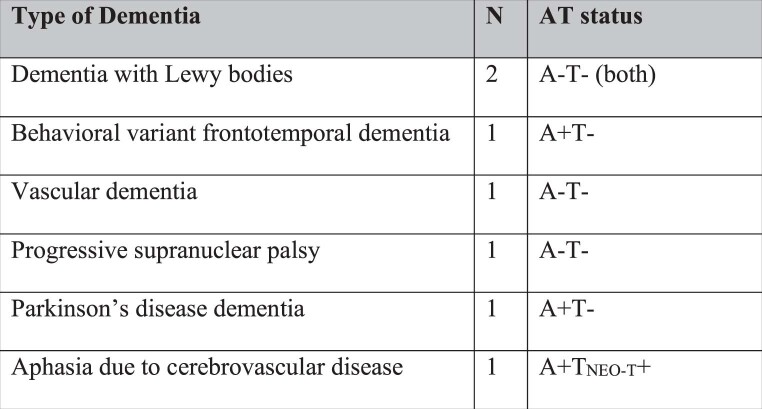
Progression to non-AD dementia types and their AT status

**Extended Data Table 4:**
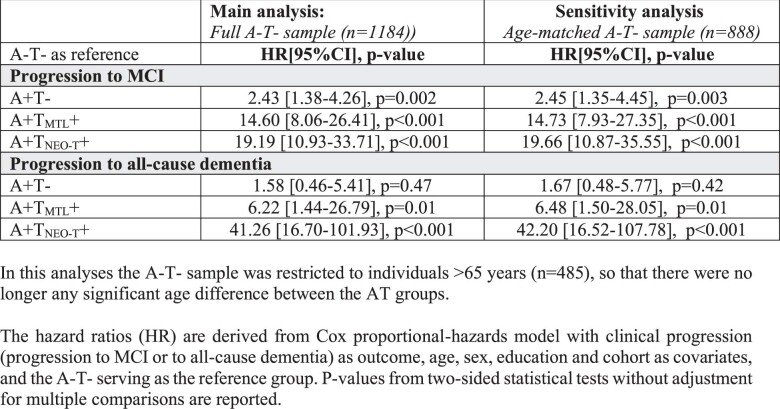
Survival analyses for the full A^-^T^-^ sample versus an age-matched A^-^T^-^ sample. In this analysis, the A^-^T^-^ sample was restricted to individuals older than 65 years (*n* = 485), so that there was no longer any significant age difference between the AT groups. The hazard ratios are derived from a Cox proportional-hazards model with clinical progression (progression to MCI or to all-cause dementia) as the outcome and age, sex, education and cohort as covariates; A^−^T^−^ served as the reference group. *P* values from two-sided statistical tests without adjustment for multiple comparisons are reported

**Extended Data Table 5:**
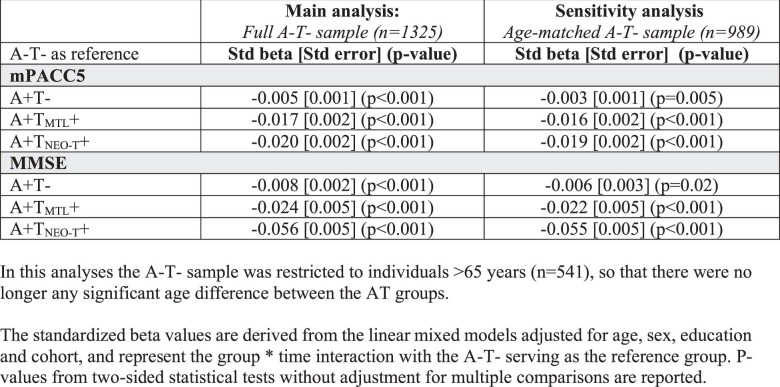
Linear mixed models for the full A^-^T^-^ sample versus an age-matched A^-^T^-^ sample. In this analysis, the A^-^T^-^ sample was restricted to individuals older than 65 years (*n* = 541), so that there was no longer any significant age difference between the AT groups. The standardized beta values are derived from the linear mixed models adjusted for age, sex, education and cohort, and represent the group × time interaction with the A^−^T^−^ serving as the reference group. *P* values from two-sided statistical tests without adjustment for multiple comparisons are reported

**Extended Data Table 6:**
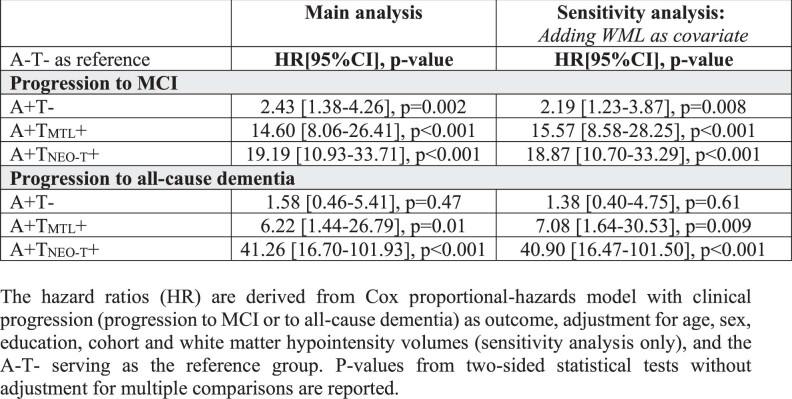
Survival analyses with and without covarying for white matter pathology. The hazard ratios are derived from a Cox proportional-hazards model with clinical progression (progression to MCI or to all-cause dementia) as the outcome, adjustment for age, sex, education, cohort and white matter hypointensity volumes (sensitivity analysis only) and A^−^T^−^ serving as the reference group. *P* values from two-sided statistical tests without adjustment for multiple comparisons are reported

**Extended Data Table 7:**
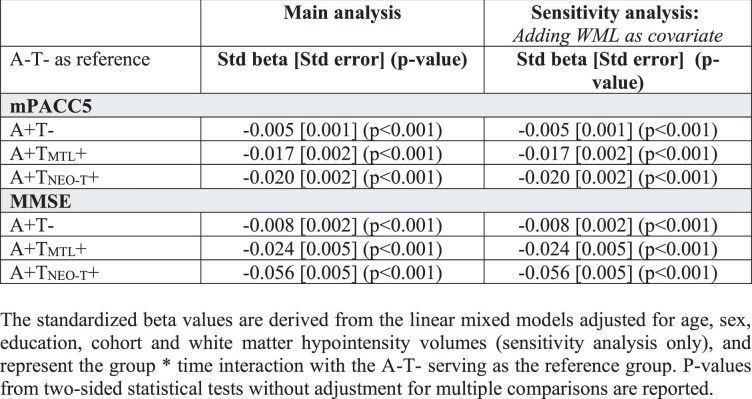
Linear mixed models with and without covarying for white matter pathology. The standardized beta values are derived from the linear mixed models adjusted for age, sex, education, cohort and white matter hypointensity volumes (sensitivity analysis only) and represent the group × time interaction with A^−^T^−^ serving as the reference group. *P* values from two-sided statistical tests without adjustment for multiple comparisons are reported

## Data Availability

Due to the multicenter design of the study, access to individual participant data from each cohort would need to be made available through the principal investigators or project websites of the respective cohorts. For the MCSA, raw and analyzed de-identified data can be requested at https://ras-rdrs.mayo.edu/Request/IndexRequest. The request will be reviewed by the MCSA investigators and Mayo Clinic to verify whether it is subject to any intellectual property or confidentiality obligations. A data sharing agreement must be obtained before release. For BioFINDER-1 and BioFINDER-2, anonymized data will be shared by request to O.H. from a qualified academic investigator for the sole purpose of replicating the procedures and results presented in the article and as long as data transfer is in agreement with European Union legislation on the general data protection regulation and decisions by the Swedish Ethical Review Authority and Region Skåne, which should be regulated in a material transfer agreement. For BACS, data are available on request to W.J.J. Requests for data from the open access part of HABS can be submitted to https://habs.mgh.harvard.edu. Requests for access to the AIBL data can submitted via an online form available at https://aibl.csiro.au/adni/index.html. For the ADC, the dataset used for the present study is available from R.O. and/or W.F. upon reasonable request.

## Code availability

The codes used for the data analyses in our study can be requested from the corresponding authors (R.O., O.H.).
