# Supplementary material for: Amyloid and tau PET-positive cognitively unimpaired individuals are at high risk for future cognitive decline
Source: Nat Med. 2022 Nov 10;28(11):2381–7. doi: 10.1038/s41591-022-02049-x (PMC9671808; doi:10.1038/s41591-022-02049-x)
Supplement: Supplementary file 1 — Supplementary Tables 1–5 and Figs. 1 and 2. [file 41591_2022_2049_MOESM1_ESM.pdf]

nature medicine

Article

https://doi.org/10.1038/s41591-022-02049-x

Amyloid and tau PET-positive cognitively unimpaired individuals are at high risk for future cognitive decline

In the format provided by the authors and unedited

SUPPLEMENTARY CONTENT

Table/Figure	Title	Page
Table 1	Participant characteristics by cohort	2
Figure 1	Baseline tau PET load for A+T_{MTL}+ and A+T_{NEO-T}+ groups per cohort	9
Table 2	Cohorts descriptions	10
Table 3	Methods to determine Amyloid PET status by cohort	12
Table 4	Methods to determine Tau PET status by cohort	14
Table 5	Composition of the mPACC5 for each cohort	15
Figure 2	Correlation between the original PACC5 and the mPACC5	16

Supplementary Table 1. Participant characteristics by cohort

COHORT 1: Mayo Clinic Olmsted Study of Aging (MCSA), n=665

	**A-T-**	**A+T-**	**A+T_{MTL}+**	**A+T_{NEO-T}+**
N	446 (67%)	173 (26%)	25 (4%)	21 (3%)
Age, years	66.8±9.2	76.4±8.3	75.1±6.7	79.5±6.3
Sex, n (%) female	209 (47%)	80 (46%)	12 (48%)	6 (29%)
Education, years	15.2±2.4	14.7±2.5	15.5±2.7	14.7±3.5
MMSE, baseline score	28.9±1.0	28.4±1.2	28.5±1.4	28.4±1.0
WM hypointensities	3498.0±5113.3	6459.3±7713.7	4824.4±4884.3	9510.0±11785.1
Follow-up duration, months	52.7±17.7	46.2±18.5	46.7±18.9	40.9±18.9
Follow-up visits, number	5.0±1.2	4.7±1.3	4.8±1.2	4.5±1.2
Progression to MCI (%)	21 (5%)	16 (9%)	11 (44%)	6 (29%)
Progression to all-cause dementia (%)	2 (0.4%)	2 (1%)	1 (4%)	1 (5%)

COHORT 2: BioFINDER-1, n=56

	**A-T-**	**A+T-**	**A+T_{MTL}+**	**A+T_{NEO-T}+**
N	29 (52%)	15 (28%)	5 (9%)	7 (13%)
Age, years	74.2±7.3)	74.7±7.4	75.6±7.1	72.6±8.9
Sex, n (%) female	13 (45%)	9 (60%)	4 (80%)	3 (43%)
Education, years	12.6±3.9	12.1±3.9	9.2±1.5	12.1±2.1
MMSE, baseline score	29.0±1.1	29.7±0.5	29.0±0.7	27.6±1.6
WM hypointensities	5296.8±4541.7	6183.6±7577.9	3264.0±1340.0	7584.4±7345.0
Follow-up duration, months	37.6±8.4	34.1±9.0	36.0±5.3	36.0±10.0
Follow-up visits, number	2.4±0.7	2.4±0.6	3.0±0.0	3.1±1.2
Progression to MCI (%)	0 (0%)	2 (13%)	1 (20%)	6 (86%)
Progression to all-cause dementia (%)	0 (0%)	1 (7%)	0 (0%)	4 (57%)

COHORT 3: BioFINDER-2, n=222

	**A-T-**	**A+T-**	**A+T$_{MTL}$+**	**A+T$_{NEO-T}$+**
N	148 (67%)	51 (23%)	10 (5%)	13 (6%)
Age, years	65.3±10.1	70.1±9.3	74.1±6.8	75.8±7.7
Sex, n (%) female	75 (51%)	23 (45%)	6 (60%)	9 (69%)
Education, years	12.6±3.3	12.6.±3.8	11.2±4.0	11.1±3.5
MMSE, baseline score	29.0±1.2	28.8±1.3	28.0±1.6	28.7±1.0
WM hypointensities	7239.6±6006.0	7913.7±6284.1	6381.4±3004.9	10378.5±8082.1
Follow-up duration, months	29.1±10.7	36.3±8.8	39.4±5.1	37.1±9.8
Follow-up visits, number	2.5±0.8	3.5±1.0	4.1±0.7	3.2±1.0
Progression to MCI (%)	3 (2%)	4 (8%)	7 (70%)	8 (62%)
Progression to all-cause dementia (%)	1 (1%)	0 (0%)	1 (10%)	4 (31%)

COHORT 4: The Berkeley Aging Cohort study (BACS), n=107

	**A-T-**	**A+T-**	**A+T$_{MTL}$+**	**A+T$_{NEO-T}$+**
N	62 (58%)	36 (34%)	4 (4%)	5 (5%)
Age, years	77.1±7.1	76.7±4.4	81.0±2.3	75.8±3.0
Sex, n (%) female	36 (58%)	23 (64%)	1 (25%)	3 (60%)
Education, years	17.4±2.9	16.7±1.9	16.2±1.3	16.2±0.8
MMSE, baseline score	28.8±1.1	28.6±1.5	27.5±1.3	28.8±0.8
WM hypointensities	7286.2±7759.8	4689.3±3998.3	4984.9±3343.9	5681.4±5583.3
Follow-up duration, months	32.2±15.7	33.6±15.2	21.6±11.4	38.0±12.4
Follow-up visits, number	3.5±1.5	3.6±1.4	2.8±0.5	3.2±1.6
Progression to MCI (%)	NA	NA	NA	NA
Progression to all-cause dementia (%)	NA	NA	NA	NA

COHORT 5: The Harvard Aging Brain Study (HABS), n=155

	**A-T-**	**A+T-**	**A+T$_{MTL}$+**	**A+T$_{NEO-T}$+**
N	103 (66%)	38 (25%)	5 (3%)	9 (6%)
Age, years	75.0±6.3	77.3± 6.3	76.8±5.7	77.4±5.3
Sex, n (%) male	56 (54%)	21 (55%)	4 (80%)	7 (78%)
Education, years	15.9±3.3	16.1±3.0	16.4±2.6	17.3±1.4
MMSE, baseline score	29.3±1.0	29.2±0.9	29.0±1.4	28.3±1.2
WM hypointensities	3385.3±2587.2	8369.5±13046.5	4868.3±5016.7	2927.1±1990.0
Follow-up duration, months	29.2±12.6	26.2±14.8	23.1±14.1	21.9±4.6
Follow-up visits, number	3.6±1.0	3.4±1.0	3.2±1.1	3.2±0.4
Progression to MCI (%)	1 (1%)	3 (8%)	2 (40%)	3 (33%)
Progression to all-cause dementia (%)	0 (0%)	0 (0%)	0 (0%)	0 (0%)

COHORT 6: The Australian Imaging Biomarkers and Lifestyle Study of Ageing (AIBL), n=46

	**A-T-**	**A+T-**	**A+T_{MTL}+**	**A+T_{NEO-T}+**
N	30 (65%)	9 (20%)	3 (7%)	4 (9%)
Age, years	72.2±5.5	78.9±8.0	84.7±0.6	76.5±6.6
Sex, n (%) male	17 (57%)	6 (67%)	2 (67%)	3 (75%)
Education, years	12.7±2.4	13.3±2.1	9.5±1.7	11.6±2.2
MMSE, baseline score	28.9±1.0	28.8±1.6	26.3±2.5	26.2±2.4
WM hypointensities	3336.8±4141.8	10398.4±17650.3	3157.7±1010.9	3035.2±2687.4
Follow-up duration, months	50.5±14.9	46.6±20.0	39.5±4.3	47.0±17.1
Follow-up visits, number	3.7±0.9	3.9±1.4	2.7±0.6	3.0±1.2
Progression to MCI (%)	1 (3%)	1 (11%)	2 (67%)	4 (100%)
Progression to all-cause dementia (%)	0 (0%)	0 (0%)	0 (0%)	2 (50%)

Education was originally recorded as categorical data, i.e., 7-8 years, 9-12 years, 13-15 years or 15+ years. To be able to include this variable with the other cohorts, we converted it to the average of each category, or to 15 for the last category.

COHORT 7: The Amsterdam Dementia Cohort (ADC), n=40

	**A-T-**	**A+T-**	**A+T_{MTL}+**	**A+T_{NEO-T}+**
N	25 (63%)	6 (15%)	3 (8%)	6 (15%)
Age, years	63.1±7.0	71.0±6.4	69.7±2.5	70.0±4.0
Sex, n (%) male	13 (52%)	2 (33%)	2 (67%)	3 (50%)
Education, years	12.5±2.6	12.5±3.8	10.0±0	13.3±3.1
WM hypointensities	2582.4±3861.5	3716.5±3843.9	1745.7±651.5	3405.1±2461.0
MMSE, baseline score	28.9±1.1	28.2±1.5	28.7±1.2	28.0±1.4
Follow-up duration, months	33.4±9.6	27.5±9.6	27.0±8.5	33.3±12.2
Follow-up visits, number	4.1±0.8	3.8±0.8	3.3±0.6	3.7±0.5
Progression to MCI (%)	0 (0%)	0 (0%)	2 (67%)	5 (83%)
Progression to all-cause dementia (%)	1 (4%)	0 (0%)	0 (0%)	1 (17%)

Supplementary Figure 1. Baseline tau PET load for A+T$_{MTL}$+ and A+T$_{NEO\text{-}T}$+ groups per cohort

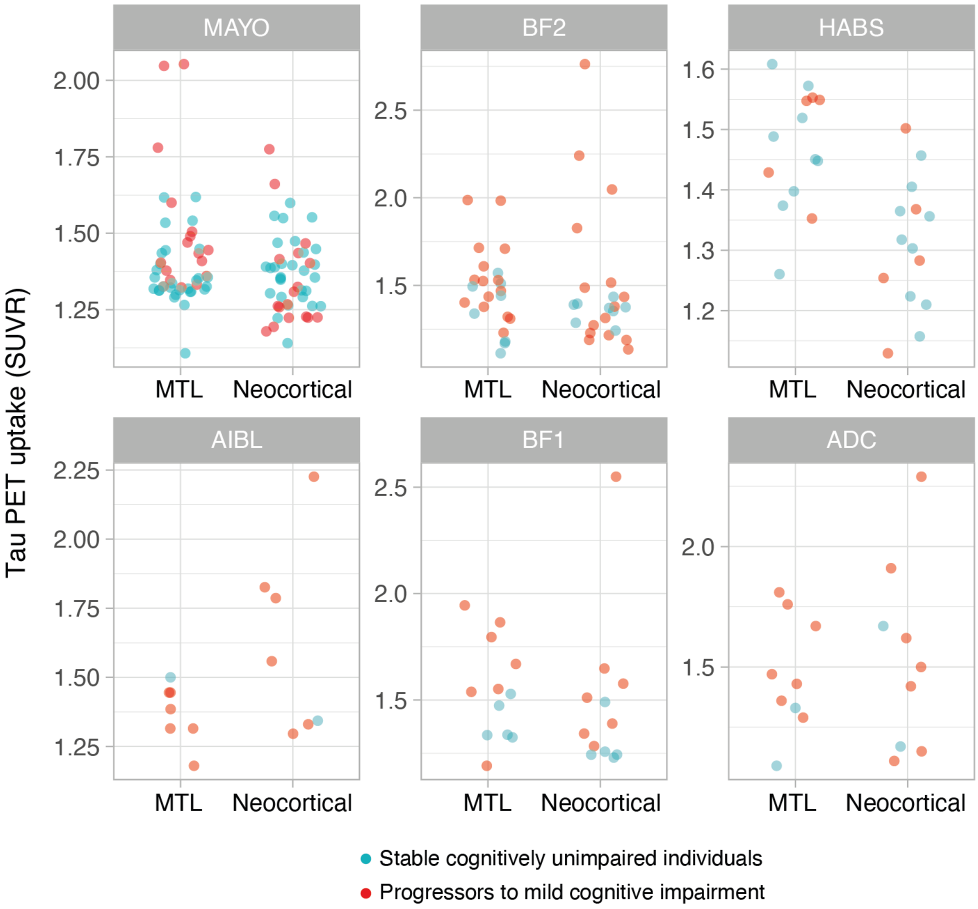

The y-axis indicates the standardized uptake value ratio within the medial temporal lobe and neocortical regions-of-interest, displayed for each cohort separately because images were not centrally analyzed. Red and blue colors indicate A+T+ CU individuals who did or did not, respectively, progress to MCI during the time course of this study.

Supplementary Table 2. Cohort descriptions

Cohort	Cohort description	References
BioFINDER-1 & BioFINDER-2	The Swedish BioFINDER studies are longitudinal studies covering the entire AD continuum in which participants were recruited at Skåne University Hospital and the Hospital of Angelholm, Sweden. The main inclusion criteria were absence of cognitive symptoms as assessed by a physician with special interest in cognitive disorders, being fluent in Swedish, having no significant unstable systemic illness that made it difficult to participate in the study, having no current significant alcohol or substance misuse, and no significant neurological or psychiatric illness. For the current study participants above > 50 years old were included. Both cognitively healthy older adults and SCD participants were included. The SCD participants were referred from participating memory clinic because of cognitive complaints, but did not fulfill criteria for MCI (defined using criteria by Petersen and operationalized according to[61,62]) following a neuropsychological test battery.	63-65
MCSA	The Mayo Clinic Study of Aging (MCSA) is a longitudinal population-based study of cognitive aging in Olmsted County, Minnesota. The study was designed to study prevalence, incidence and risk factors for MCI and dementia. Potential participants are randlomly enumerated from the Olmsted County, MN, census and enrolled by age/sex strata. Enumeration is repeated to maintain a sample of approximately 3000 active participants. At entry, every person underwent evaluations that included a medical history review and interview with the participant and a study partner, a neurological examination by a physician; and a neuropsychological examination. For this study, participants were considered MCI only if the study coordinator, physician, and neuropsychologist were all in agreement regarding the MCI diagnosis. Participants were judged cognitively normal if they did not meet MCI criteria. Participants aged between 50 and 89 years old were included in the current study.	66
BACS	The Berkeley Aging Cohort Study (BACS) is a community-dwelling cohort that is a convenience sample of healthy individuals who are older than 60 years and reside in the San Francisco Bay Area of California. Inclusion criteria were no impairment of activities of daily living, absence of any neurological or psychiatric condition that potentially affects brain structure and function, no cognitive complaints, normal perform ance on cognitive tests (maximally 1.5 standard deviation below age-, education-, and sex-adjusted norms), no use of psychoactive drugs and absence of sensory impairment that might interfere with cognitive testing.	67

	MCI or dementia diagnosis was not available in BACS and thus this cohort was included in analyses of cognitive trajectories only.	
HABS	The Harvard Aging Brain Study (HABS) is a a longitudinal study on aging and AD, from Memory Disorders Clinics at the Massachusetts General and Brigham and Women's Hospitals, and from the Massachusetts Alzheimer's Disease Research Center. The cohort includes cognitively normal, healthy older individuals according to the following criteria. Inclusion criteria: 65 years of age or older, a CDR score of 0, MMSE>25, scores above age- and education-adjusted norms on the 30-Minute Delayed Recall of the Logical Memory Story A, and a score of less than 11 on the Geriatric Depression Scale. Exclusion criteria: history of alcoholism, drug abuse, head trauma, or current serious medical/psychiatric illness. Data was obtained in March 2022 from data release 2.0 via https://habs.mgh.harvard.edu	68
AIBL	The Australian Imaging, Biomarker & Lifestyle Flagship Study of Ageing (AIBL) is a longitudinal, prospective cohort with participants coming from two-site study – Melbourne and Perth. To be included in the study, participants were (1) ≥60 years old; (2) fluent in English; (4) had completed at least 7 years of education; (5) did not have any history of neurological or psychiatric disorders, drug or alcohol abuse or dependence, or any other unstable medical condition; and (6) were deemed to be cognitively unimpaired (CU), based on their performance on a battery of cognitive assessments that AIBL participants undergo every 12 to 18 months. A multidisciplinary clinical review panel determines whether an individual is CU, based on the available clinical and neuropsychological information.	69,70
ADC	The Amsterdam Dementia Cohort (ADC) is a prospective cohort study including patients with subjective cognitive decline (SCD) presenting at the Alzheimer Center of the VU University Medical Center Amsterdam. All participants have been referred to the memory clinic by their general practitioner, and a neurologist or geriatrician in the case of a second opinion for evaluation of cognitive complaints. They receive standardized dementia screening at the memory clinic, including an interview with a neurologist, physical and neurological examination, neuropsychological assessment. The main inclusion criteria were a diagnosis of SCD (i.e., cognitive complaints and normal cognition) and age ≥ 45 years. Exclusion criteria are MCI, dementia, major psychiatric disorder (i.e., current depression, personality disorders, schizophrenia), neurological diseases known to cause memory complaints (i.e., Parkinson's disease, epilepsy), HIV, abuse of alcohol or other sub- stances, and language barrier.	71

Supplementary Table 3. Methods to determine Amyloid PET status by cohort

Cohort	Tracer	Methodology	Cut-off	References
BioFINDER-1	[^{18}F]flutemetamol	Global neocortical composite standardized uptake value ratios (SUVR) in bilateral prefrontal, parietal, lateral temporal, sensorimotor, occipital, and mesial temporal cortices, the anterior cingulate, posterior cingulate and precuneus for the 90-110min interval p.i. with whole cerebellum as reference region.	>1.03 SUVR	[72,73]
BioFINDER-2	[^{18}F]flutemetamol	Global neocortical composite SUVR in bilateral prefrontal, parietal, lateral temporal, sensorimotor, occipital, and mesial temporal cortices, the anterior cingulate, posterior cingulate and precuneus for the 90-110min interval p.i. with whole cerebellum as reference region	>1.03 SUVR	[72,73]
MCSA	[^{11}C]PIB	Late uptake amyloid PET images were acquired from 40-60 minutes p.i. A meta-ROI was calculated as the voxel-number weighted average of uptake in a target region including prefrontal, orbitofrontal, parietal, temporal, anterior and posterior cingulate, and precuneus regions divided by the uptake in the cerebellar crus gray matter.	>1.48 SUVR (>21CL)	[74]
BACS	[^{11}C]PIB	Distribution volume ratio (DVR) images in a global ROI encompassing frontal, temporal, parietal and anterior, and posterior cingulate cortices were calculated with Logan graphical analysis over 35–90 min data and normalized to a cerebellar gray reference region.	>1.065 DVR	[75]

HABS	[^{11}C]PIB	Global neocortical composite DVR including frontal, lateral temporal, and retrosplenial cortices from a 60-minute dynamic acquisition p.i. with cerebellar gray matter as reference tissue.	>1.2 DVR (>26 CL)	[76,77]
AIBL	[^{11}C]PIB/ [^{18}F]NAV4694	The standard Centiloid (CL) cortical (i.e., frontal, temporal and parietal cortices, precuneus, anterior striatum and insular cortex) and whole cerebellar volumes of interest template were applied to the summed and spatially normalised PET images in order to obtain SUVR's. These SUVR were transformed into CL units by linear transformation using the PET tracer-specific equations published for conversion of CL method SUVR to CL units.	>24 CL	[78]
ADC	[^{18}F]florbetapir	Visual read following guidelines provided by Avid Radiopharmaceuticals corresponding to >17 CL.	-	[79,80]

CL = Centiloid; DVR = Distribution volume ratio; SUVR = Standardized uptake value ratio.

Centiloid (CL) units were presented when available.

Supplementary Table 4. Methods to determine Tau PET status in the medial temporal lobe (MTL) and neocortex (NEO-T) by cohort

Cohort	Tracer	Scanning interval	Reference region	Reference	Cut-off MTL	Cut-off NEO-T
BioFINDER-1	[^{18}F]flortaucipir	80-100min p.i.	Inferior cerebellar GM	63	1.26 SUVR	1.29 SUVR
BioFINDER-2	[^{18}F]RO948	70-90min p.i.	Inferior cerebellar GM	65	1.34 SUVR	1.36 SUVR
MCSA	[^{18}F]flortaucipir	80-100min p.i.	Cerebellar crus GM	74	1.30 SUVR	1.37 SUVR
BACS	[^{18}F]flortaucipir	80-100min p.i.	Inferior cerebellar GM	81	1.36 SUVR	1.32 SUVR
HABS	[^{18}F]flortaucipir	80-100min p.i.	Cerebellar GM	76	1.36 SUVR	1.28 SUVR
AIBL	[^{18}F]flortaucipir	80-100min p.i.	Cerebellar GM	82	1.31 SUVR	1.38 SUVR
ADC	[^{18}F]flortaucipir	80-100min p.i.	Cerebellar GM	83	1.26 SUVR	1.26 SUVR

GM = Gray matter; MTL = Medial temporal lobe; NEO-T = Temporal Neocortical; p.i. = Post-injection; SUVR = Standardized uptake value ratio.

The cut-offs were generated in each individual cohort, based on the mean + 2*standard deviation across all Aβ-negative participants within each cohort. We computed tau PET status for a medial temporal lobe (MTL; unweighted average of bilateral entorhinal cortex and amygdala) and a neocortical (NEO-T; weighted average of bilateral middle temporal and inferior temporal gyri) region-of-interest.

Supplementary Table 5. Composition of the mPACC5 for each cohort

Cohort	Global Cognition	Episodic Memory	Timed executive function	Semantic memory
BioFINDER-1	MMSE	ADAS-COG delayed word recall	Symbol digit modalities test	Animal fluency
BioFINDER-2	MMSE	ADAS-COG delayed word recall	Symbol digit modalities test	Animal fluency
MCSA	MMSE[a]	AVLT delayed recall	WAIS-R Digit Symbol	Sum of animal, fruits and vegetables fluency
BACS	MMSE	CVLT – Delayed recall	Symbol digit modalities test	Animal fluency
HABS	MMSE	SRT – Delayed recall	Symbol digit modalities test	Animal fluency
AIBL	MMSE	CVLT – Delayed recall	Symbol digit modalities test	Sum of animal and names fluency
ADC	MMSE	RAVLT – Delayed recall	TMT-B	Animal fluency

Note that the episodic memory test was given double weight and thus accounted for 40% of the mPACC5 score.

[a] A 38-point test, the Short Test of Mental Status (STMS)[84], was converted to MMSE scores using an in-house developed algorithm[85].

Supplementary Figure 2. Correlation plot between the original PACC5 and the modified PACC5 in HABS

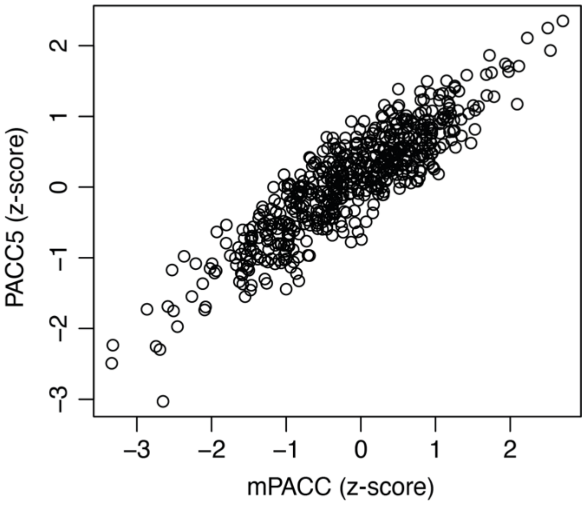

Correlation analysis between the original PACC5[86] (y-axis) and the modified version of the PACC5 used in the present study (x-axis) in the HABS cohort showed excellent overlap (r=0.89, p<0.001) between the two PACC5 versions.

61. Palmqvist, S., *et al.* Discriminative Accuracy of Plasma Phospho-tau217 for Alzheimer Disease vs Other Neurodegenerative Disorders. *JAMA* **324**, 772-781 (2020).
62. Petrazzuoli, F., *et al.* Brief Cognitive Tests Used in Primary Care Cannot Accurately Differentiate Mild Cognitive Impairment from Subjective Cognitive Decline. *Journal of Alzheimer's disease : JAD* **75**, 1191-1201 (2020).
63. Ossenkoppele, R., *et al.* Associations between tau, Abeta, and cortical thickness with cognition in Alzheimer disease. *Neurology* **92**, e601-e612 (2019).
64. Palmqvist, S., *et al.* Prediction of future Alzheimer's disease dementia using plasma phospho-tau combined with other accessible measures. *Nat Med* (2021).
65. Leuzy, A., *et al.* Diagnostic Performance of RO948 F 18 Tau Positron Emission Tomography in the Differentiation of Alzheimer Disease From Other Neurodegenerative Disorders. *JAMA Neurol* **77**, 955-965 (2020).
66. Roberts, R.O., *et al.* The Mayo Clinic Study of Aging: design and sampling, participation, baseline measures and sample characteristics. *Neuroepidemiology* **30**, 58-69 (2008).
67. Ossenkoppele, R., *et al.* Is verbal episodic memory in elderly with amyloid deposits preserved through altered neuronal function? *Cereb Cortex* **24**, 2210-2218 (2014).
68. Dagley, A., *et al.* Harvard Aging Brain Study: Dataset and accessibility. *Neuroimage* **144**, 255-258 (2017).
69. Ellis, K.A., *et al.* The Australian Imaging, Biomarkers and Lifestyle (AIBL) study of aging: methodology and baseline characteristics of 1112 individuals recruited for a longitudinal study of Alzheimer's disease. *Int Psychogeriatr* **21**, 672-687 (2009).
70. Fowler, C., *et al.* Fifteen Years of the Australian Imaging, Biomarkers and Lifestyle (AIBL) Study: Progress and Observations from 2,359 Older Adults Spanning the Spectrum from Cognitive Normality to Alzheimer's Disease. *J Alzheimers Dis Rep* **5**, 443-468 (2021).
71. Slot, R.E.R., *et al.* Subjective Cognitive Impairment Cohort (SCIENCe): study design and first results. *Alzheimers Res Ther* **10**, 76 (2018).
72. Palmqvist, S., *et al.* Accuracy of brain amyloid detection in clinical practice using cerebrospinal fluid beta-amyloid 42: a cross-validation study against amyloid positron emission tomography. *JAMA Neurol* **71**, 1282-1289 (2014).
73. Lundqvist, R., *et al.* Implementation and validation of an adaptive template registration method for 18F-flutemetamol imaging data. *J Nucl Med* **54**, 1472-1478 (2013).
74. Jack, C.R., Jr., *et al.* Defining imaging biomarker cut points for brain aging and Alzheimer's disease. *Alzheimers Dement* **13**, 205-216 (2017).
75. Villeneuve, S., *et al.* Existing Pittsburgh Compound-B positron emission tomography thresholds are too high: statistical and pathological evaluation. *Brain* **138**, 2020-2033 (2015).
76. Johnson, K.A., *et al.* Tau positron emission tomographic imaging in aging and early Alzheimer disease. *Ann Neurol* **79**, 110-119 (2016).
77. Farrell, M.E., *et al.* Defining the Lowest Threshold for Amyloid-PET to Predict Future Cognitive Decline and Amyloid Accumulation. *Neurology* **96**, e619-e631 (2021).
78. Amadoru, S., *et al.* Comparison of amyloid PET measured in Centiloid units with neuropathological findings in Alzheimer's disease. *Alzheimers Res Ther* **12**, 22 (2020).
79. Timmers, T., *et al.* Amyloid PET and cognitive decline in cognitively normal individuals: the SCIENCe project. *Neurobiol Aging* **79**, 50-58 (2019).
80. Collij, L.E., *et al.* Visual assessment of [(18)F]flutemetamol PET images can detect early amyloid pathology and grade its extent. *Eur J Nucl Med Mol Imaging* **48**, 2169-2182 (2021).
81. Scholl, M., *et al.* PET Imaging of Tau Deposition in the Aging Human Brain. *Neuron* **89**, 971-982 (2016).
82. Groot, C., *et al.* Mesial temporal tau is related to worse cognitive performance and greater neocortical tau load in amyloid-beta-negative cognitively normal individuals. *Neurobiol Aging* **97**, 41-48 (2021).

83. Timmers, T., *et al.* Associations between quantitative [(18)F]flortaucipir tau PET and atrophy across the Alzheimer's disease spectrum. *Alzheimers Res Ther* **11**, 60 (2019).
84. Kokmen, E., Smith, G.E., Petersen, R.C., Tangalos, E. & Ivnik, R.C. The short test of mental status. Correlations with standardized psychometric testing. *Archives of neurology* **48**, 725-728 (1991).
85. Tang-Wai, D.F., *et al.* Comparison of the short test of mental status and the mini-mental state examination in mild cognitive impairment. *Archives of neurology* **60**, 1777-1781 (2003).
86. Papp, K.V., Rentz, D.M., Orlovsky, I., Sperling, R.A. & Mormino, E.C. Optimizing the preclinical Alzheimer's cognitive composite with semantic processing: The PACC5. *Alzheimers Dement (N Y)* **3**, 668-677 (2017).
