# Supplementary material for: Amyloid and tau PET-positive cognitively unimpaired individuals are at high risk for future cognitive decline
Source: Nat Med. 2022 Nov 10;28(11):2381–7. doi: 10.1038/s41591-022-02049-x (PMC9671808; doi:10.1038/s41591-022-02049-x)
Supplement: Supplementary file 2 — Reporting Summary [file 41591_2022_2049_MOESM2_ESM.pdf]

nature research

Corresponding author(s): Rik Ossenkoppele & Oskar Hansson

Last updated by author(s): August 29th 2022

Reporting Summary

Nature Research wishes to improve the reproducibility of the work that we publish. This form provides structure for consistency and transparency in reporting. For further information on Nature Research policies, see our Editorial Policies and the Editorial Policy Checklist.

Statistics

For all statistical analyses, confirm that the following items are present in the figure legend, table legend, main text, or Methods section.

n/a	Confirmed	
☐	☒	The exact sample size (*n*) for each experimental group/condition, given as a discrete number and unit of measurement
☐	☒	A statement on whether measurements were taken from distinct samples or whether the same sample was measured repeatedly
☐	☒	The statistical test(s) used AND whether they are one- or two-sided<br>*Only common tests should be described solely by name; describe more complex techniques in the Methods section.*
☐	☒	A description of all covariates tested
☐	☒	A description of any assumptions or corrections, such as tests of normality and adjustment for multiple comparisons
☐	☒	A full description of the statistical parameters including central tendency (e.g. means) or other basic estimates (e.g. regression coefficient) AND variation (e.g. standard deviation) or associated estimates of uncertainty (e.g. confidence intervals)
☐	☒	For null hypothesis testing, the test statistic (e.g. *F*, *t*, *r*) with confidence intervals, effect sizes, degrees of freedom and *P* value noted<br>*Give P values as exact values whenever suitable.*
☒	☐	For Bayesian analysis, information on the choice of priors and Markov chain Monte Carlo settings
☒	☐	For hierarchical and complex designs, identification of the appropriate level for tests and full reporting of outcomes
☐	☒	Estimates of effect sizes (e.g. Cohen's *d*, Pearson's *r*), indicating how they were calculated

Our web collection on statistics for biologists contains articles on many of the points above.

Software and code

Policy information about availability of computer code

Data collection	No software was used.
Data analysis	R version 4.0.5 was used for all analyses.

For manuscripts utilizing custom algorithms or software that are central to the research but not yet described in published literature, software must be made available to editors and reviewers. We strongly encourage code deposition in a community repository (e.g. GitHub). See the Nature Research guidelines for submitting code & software for further information.

Data

Policy information about availability of data

All manuscripts must include a data availability statement. This statement should provide the following information, where applicable:

- Accession codes, unique identifiers, or web links for publicly available datasets
- A list of figures that have associated raw data
- A description of any restrictions on data availability

Due to the multicentric design of the study, access to individual participant data from each cohort would have to be made available through the PIs of the respective cohorts. Generally, anonymised data can be shared by request from qualified academic investigators for the purpose of replicating procedures and results presented in the article, as long as data transfer is in agreement with the data protection regulation at the institution and is approved by the local Ethics Review Board. Requests for data from the open-access part of HABS can be submitted to: https://habs.mgh.harvard.edu.

Field-specific reporting

Please select the one below that is the best fit for your research. If you are not sure, read the appropriate sections before making your selection.

☒ Life sciences ☐ Behavioural & social sciences ☐ Ecological, evolutionary & environmental sciences

For a reference copy of the document with all sections, see nature.com/documents/nr-reporting-summary-flat.pdf

Life sciences study design

All studies must disclose on these points even when the disclosure is negative.

Sample size	We did not a priori perform a sample size calculation for this study. Given the relative rarity of amyloid (A) and tau (T) PET positive cognitively unimpaired individuals. we aimed to maximize the sample size of especially the A+T+ individuals by pooling data across 7 different cohorts. We used the following inclusion criteria: All participants were i) cognitively unimpaired at baseline defined by neuropsychological test scores within the normative range given an individuals' age, sex and educational background, ii) had amyloid PET available to determine amyloid status, iii) underwent a tau PET scan before January 1, 2019, to allow for sufficiently long follow-up duration, and iv) had at least one clinical follow-up visit available. Based on previous studies showing strong predictive value of tau PET on cognitive decline, the total sample sizes of 55 A+Tmtl+ and 65 A+Tneo+ participants was deemed sufficient to demonstrate significant differences in rates of clinical progression relative to A+T- and A-T- groups.
Data exclusions	No data were excluded from the analysis.
Replication	We pooled all data obtained from the cohorts. Consequently, we did not replicate the findings in the separate cohorts, but we do show all cohort-specific characteristics in the Extended Data.
Randomization	Based on amyloid and tau PET status we generated four different biomarker groups: A-T-, A+T-, A+TMTL+ (defined as tau PET positive in the MTL but not in the neocortex) and A+TNEO+ (defined as tau PET positive in the neocortex).
Blinding	PET analyses were performed by individuals who were blinded to the clinical data. Authors who performed the data preprocessing were blinded to demographic and clinical characteristics of individuals.

Reporting for specific materials, systems and methods

We require information from authors about some types of materials, experimental systems and methods used in many studies. Here, indicate whether each material, system or method listed is relevant to your study. If you are not sure if a list item applies to your research, read the appropriate section before selecting a response.

Materials & experimental systems

n/a	Involved in the study	
☒	☐	Antibodies
☒	☐	Eukaryotic cell lines
☒	☐	Palaeontology and archaeology
☒	☐	Animals and other organisms
☐	☒	Human research participants
☐	☒	Clinical data
☒	☐	Dual use research of concern

Methods

n/a	Involved in the study	
☒	☐	ChIP-seq
☒	☐	Flow cytometry
☒	☐	MRI-based neuroimaging

Human research participants

Policy information about studies involving human research participants

Population characteristics	Detailed information is given in Table 1 for all cohorts combined and in Extended Table 1 for each individual cohort.
Recruitment	Detailed information about the recruitment strategies is provided in Extended Table 4 for each individual cohort.
Ethics oversight	Local institutional review boards for human research approved the study. This includes the Mayo Clinic and Olmsted Medical Center Institutional Review Boards for MSCA, the regional Ethics Committee at Lund University for BioFINDER-1 and BioFINDER-2, the Institutional Review Board at Lawrence Berkeley National Laboratory and the University of California, Berkeley, for BACS, the Institutional Human Research Ethics Committees of Austin Health, St. Vincent's Health, Hollywood Private Hospital and Edith Cowan University for AIBL, the Partners Human Research Committee for HABS, and the Medical Ethics Review Committee of the Amsterdam University Medical Center for ADC.

Note that full information on the approval of the study protocol must also be provided in the manuscript.

Clinical data

Policy information about clinical studies

All manuscripts should comply with the ICMJE guidelines for publication of clinical research and a completed CONSORT checklist must be included with all submissions.

Clinical trial registration	BioFINDER-1: NCT01208675, BioFINDER-2: NCT03174938.
Study protocol	MSCA: https://www.mayo.edu/research/clinical-trials/cls-20311806<br>BioFINDER-1: https://clinicaltrials.gov/ct2/show/NCT01208675<br>BioFINDER-2: www.biofinder.se<br>AIBL: https://aibl.csiro.au/<br>ADC: https://www.alzheimercentrum.nl/wetenschap/amsterdam-dementia-cohort/<br>BACS: https://jagustlab.neuro.berkeley.edu/bacs<br>HABS: https://habs.mgh.harvard.edu/
Data collection	Data were collected between Jan 1 2014 and April 1 2022. Participating cohorts included MSCA (a population-based cohort in Rochester, USA), BioFINDER-2 and BioFINDER-2 (a mix of population-based and memory clinic-based studies in Lund and Malmo, Sweden), BACS (a population-based cohort in Berkeley, USA), AIBL (a multicenter study that combines population-based and memory clinic-based participants from Australia), HABS (a population-based cohort in Boston, USA) and the ADC (a memory clinic-based sample from Amsterdam, the Netherlands).
Outcomes	The predefined primary outcome measures are 1) progression to MCI or all-cause dementia and 2) longitudinal changes on the mPACC5 and MMSE. As predefined secondary outcomes we assessed the mPACC5 subcomponents (i.e., delayed episodic memory, timed executive functions and semantic memory).
